# Optical sensitivities of current gravitational wave observatories at higher kHz, MHz and GHz frequencies

**DOI:** 10.1038/s41598-025-08668-x

**Published:** 2025-07-16

**Authors:** Roman Schnabel, Mikhail Korobko

**Affiliations:** Institut für Quantenphysik & Zentrum für Optische Quantentechnologien, Universität Hamburg, Luruper Chaussee 149, 22761 Hamburg, Germany

**Keywords:** Gravitational wave detection, High-frequency gravitational wave, Free spectral range, Antennae pattern, GEO 600, KAGRA, LIGO, Virgo, Astronomical instrumentation, Quantum optics, General relativity and gravity

## Abstract

GEO 600, KAGRA, LIGO, and Virgo were built to observe gravitational waves at frequencies in the audio band, where the highest event rates combined with the largest signal to noise ratios had been predicted. Currently, hypothetical sources of cosmological origin that could have produced signals at higher frequencies are under discussion. Despite relevant previous research by other authors, it is not widely known that the current interferometric GW observatories have a frequency comb of high optical sensitivity that encompasses these high frequencies. Here we calculate the high-frequency noise spectral densities of operating GW observatories under the justified assumption that photon shot noise is the dominant noise source. We explain the underlying physics of why high sensitivity is achieved for all integer multiples of the free spectral ranges of the observatory’s resonators when an interferometer arm is not orientated perpendicular to the propagation direction of the GW. Proposals for new concepts of high-frequency GW detectors must be compared with the high-frequency sensitivities presented here.

## Introduction

At the time when Rainer Weiss analysed the concept of earthbound laser interferometric gravitational wave (GW) detection in terms of signal strength and noise more than 50 years ago^1^, astrophysical sources of signals in the audio band were known^2,3^. The probability of being able to measure GW signals on Earth in this band increased further over the following ten years^4–8^. At the turn of the millennium, a total of six Michelson-type laser-interferometric GW detectors – GEO 600^9^, LIGO (3)^10^, TAMA^11^ and Virgo^12^ – were under construction, targeting the audio-band. On September 14th, 2015, Advanced LIGO observed the first GW, which had frequency components up to about 300 Hz, emitted by the merger of two black holes at a distance of about 1.3 billion light years^13^. By 2020, up to 90 signals from compact binary mergers were detected by LIGO and Virgo^14^.

Also *below* the audio band, a large number of sources are expected to emit signals of measurable amplitude. Avoiding the strong terrestrial noise in this frequency range, LISA is a space-based GW observatory that targets the range from 0.1 mHz to 0.1 Hz^15^. It is due to be launched in the 2030s. Pulsar timing arrays (PTAs) are used to measure GW in the nHz range^16^. In 2023, several PTA collaborations found evidence for an incoherent background of gravitational waves produced by the collisions and mergers of supermassive black holes, see for instance^17^.

**Figure 1:**
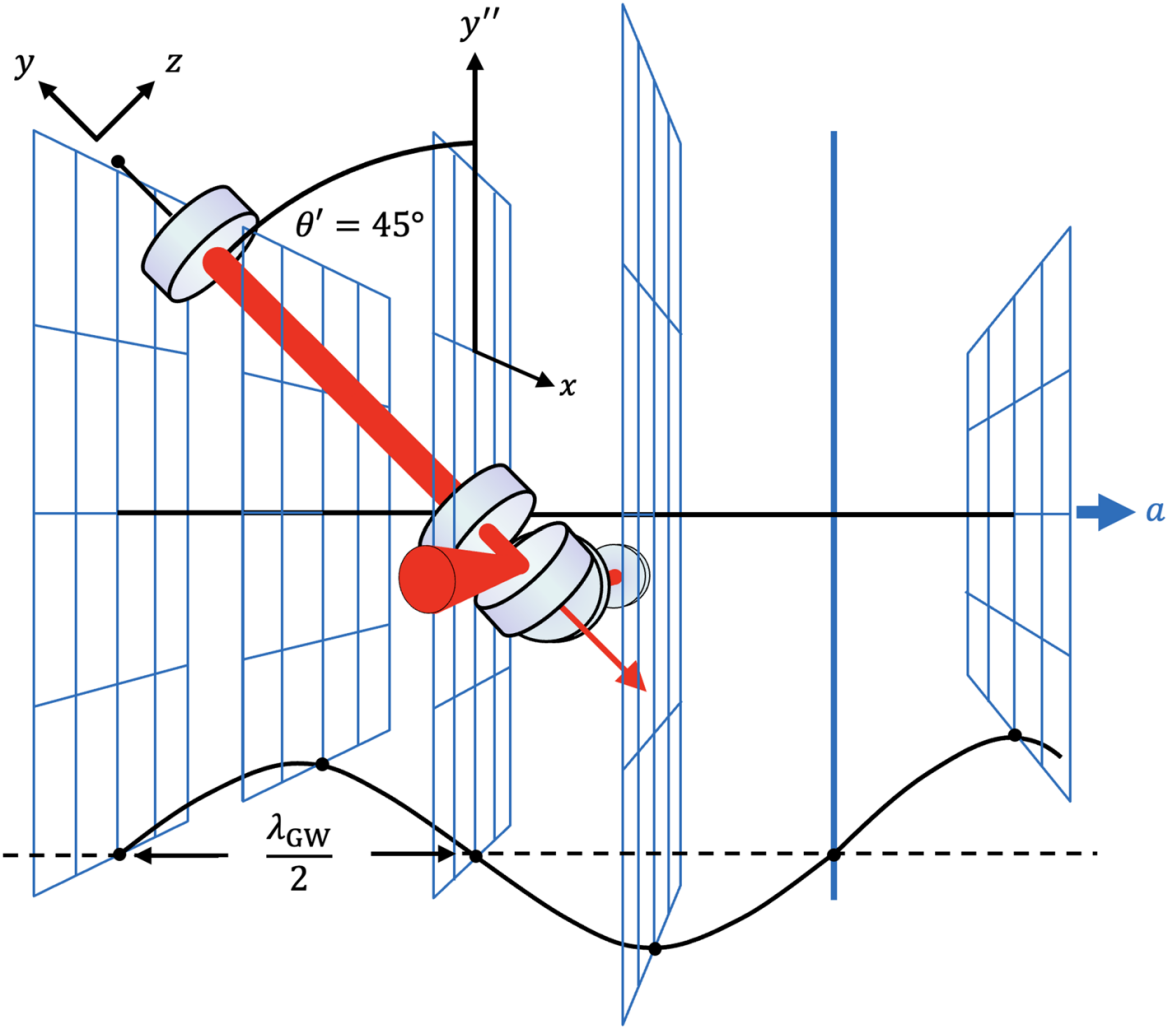
Illustration of an example alignment for efficiently observing a high-frequency GW propagating along *a* and having a wavelength that corresponds to the round trip length of the arm resonators of an interferometric GW observatory ($\lambda _\textrm{GW} \!=\! 2L$). Here, the arm resonator along the *x*-direction does not sense any signal. The arm resonator tilted by $\theta = 45^\circ$, however, does. While the optical properties of the arm resonator remain unchanged, the angle reduces the projection onto the $y''$-axis by a factor of $\sqrt{2}$. The different scaling prevents the complete cancellation of the optical phase signal, which is then resonantly amplified by the resonator. Image construction by RS. Drawing with PowerPoint version 16.85.

GWs at frequencies *above* the audio band would be rather exotic. There are no known astrophysical sources from star formation or evolution that could emit such high frequencies with measurable amplitudes. However, cosmological sources from the early universe cannot be ruled out. The early universe is not well understood, and inflation^18^, first-order phase transitions^19^, topological defects^20^, and other effects could have generated gravitational waves that still have frequencies in the MHz or even GHz range today despite redshift. A review can be found in Ref. ^21^. New detectors have been proposed to measure gravitational waves above 10 kHz. A recent and highly cited review is given by Ref. ^22^. However, the authors largely overlooked that today’s gravitational wave detectors can measure high-frequency gravitational waves not only via the non-linear memory effect^23,24^, but also have a considerable optical sensitivity above 10 kHz as well as in the MHz and GHz range. Previous research already showed in 2002 that first-generation GW detectors have relatively high sensitivity at frequencies corresponding to integer multiples of the free spectral range (FSR) of the arm resonators^25,26^. In the case of the LIGO arm resonators, the FSR is 37.5 kHz^27^. A readout channel for this frequency was developed by down-converting the signal to match the existing LIGO data acquisition system^28^. Antenna pattern was analyzed on gravitational-wave polarisation and frequencies corresponding to multiples of the cavity free spectral range^29,30^.

Here, we present the optical strain sensitivities up to $10^{11}$ Hz of the Advanced LIGO observatory, representing also Virgo and KAGRA^31^, as well as GEO 600. We additionally consider a 100-m and a 1-m laser interferometer with high-finesse arm resonators, but no other resonant enhancements such as power or signal recycling. At about 1 MHz, current LIGO achieves a one-sided strain-normalised shot noise spectral density of the order of $10^{-22}/\sqrt{\rm Hz}$. Generally, such high sensitivities at high frequencies are achieved if observatory arm resonators are tilted in the direction of GW propagation and if the GW frequency matches the differential frequency of two longitudinal modes of the optical resonator. The latter condition corresponds to the situation when the arm resonator roundtrip length equals an integer multiple of the GW wavelength. An example is illustrated in Fig. 1.

## Metric of a weak gravitational wave

In all metrics that solve Einstein’s field equations, the 4-dimensional events along a propagating laser beam have zero distances. The metric of a weak gravitational wave that is $(+)$polarised and propagates along the z-direction is therefore described in the transverse-traceless (TT) gauge by

1 $$\begin{aligned} ds^2 \!=\! -c^2 dt^2 + (1\!+\!h_+) dx^2 + (1\!-\!h_+) dy^2 + dz^2\!=0 \, , \end{aligned}$$

where $|h_+| \!\ll \! 1$ is the amplitude of the polarised GW as described above and *c* is the speed of light. Eq. (1) makes it possible to easily determine the change in the propagation time of a laser beam along the *y*-direction (or *x*-direction) ^32–34^. The above equation simplifies to

2 $$\begin{aligned} c^2 dt^2 = (1 - h_+(t,y))dy^2 \, . \end{aligned}$$

The time span $\tau (y)$ that the light needs to propagate from $y_0$ to *y* is for $|h_+| \!\ll \! 1$ then given by

3 $$\begin{aligned} \tau (y)&= t_0 + \frac{1}{c}\int \limits _{y_0}^{y} \sqrt{1-h_+ (\tau _0(y'))} \;dy' \nonumber \\&\approx t_0 + \frac{y-y_0}{c} - \frac{1}{2c}\int \limits _{y_0}^{y} h_+\!\left( \tau _0(y')\right) dy' \, , \end{aligned}$$

where $\tau _0(y') = t+(y'-y_0)/c$ is the unperturbed time span for light starting at time *t*. For a monochromatic GW with amplitude $h_+(t) \!=\! h_+ \!\cos (2\pi f t)$ and a laser beam that is retro-reflected by a mirror at distance *L*, the change in the round trip time at $y_0=0$ is given by

4 $$\begin{aligned} \Delta \tau _{2L}(t) = -\frac{h_{+}}{2c}\int \limits _0^{L} \cos \!\left[ 2\pi f {\left( \!t - \frac{2 L}{c}+\frac{y'}{c}\right) } \right] dy' + \frac{h_{+}}{2c}\int \limits _L^0 \cos \!\left[ 2\pi f {\left( \!t+\frac{y'}{c}\right) } \right] dy' \, . \end{aligned}$$

The solution to the integral after performing the Laplace transform provides the known amplitude for the change in the round-trip time in an arm resonator aligned along *y*-axis in case of a $(+)$polarized GW,

5 $$\begin{aligned} |\Delta \tau _{2L}(f)| = \frac{Lh_+}{c} {{\,\textrm{sinc}\,}}\! \left( \frac{\pi f}{f_\textrm{FSR}}\right) \, , \end{aligned}$$

where $f_\textrm{FSR} = c/(2L)$ is the free spectral range of the arm resonator. For an arm resonator in the *x* direction, Eq. (5) has the opposite sign. For low GW frequencies, i.e. $f \!\ll \! f_\textrm{FSR}$ (audio-band frequencies for km-scale arm resonators) one gets the well-known relation

6 $$\begin{aligned} |\Delta \tau _{2L}| \approx \frac{Lh_+}{c} \;\;\;\Rightarrow \;\;\; \Delta L \approx \frac{Lh_+}{2} \, , \end{aligned}$$

where $\Delta L$ is the amplitude of the effective arm length change.

Relevant for this work is Eq. (5). It states that the time delay shows local maxima, if the GW frequency is $f = (n-0.5) \!\cdot \! f_\textrm{FSR}$, with *n* a natural number greater than zero. Unfortunately, precisely these signal frequencies are off-resonant and are maximally suppressed in the arm cavities. Eq. (5) further states that the time delay due to GW is zero, if the GW frequency corresponds to the distance to one of the higher longitudinal resonance frequencies, i.e. $f = n \!\cdot \! f_\textrm{FSR}$. Often overlooked, however, is the limited range of validity of Eq. (5), and it is therefore wrongly concluded that laser interferometric GW observatories are generally not sensitive to GWs at these “FSR-frequencies”. In fact, the current GW observatories are only insensitive to these high-frequency gravitational waves if they come from the zenith (or nadir). For all other alignments, interferometric GW observatories have significant response precisely at all GW frequencies that correspond to an integer multiple of $f_\textrm{FSR}$. The rather high response at these particular frequencies comes from the fact that optical resonators resonantly enhance all signal frequencies $f = n \!\cdot \! f_\textrm{FSR}$ since these frequencies correspond to the frequency separation of neighbouring longitudinal resonator modes.

As an example, we calculate the time delay for a resonator round trip when the resonator is inclined at $\theta = 45^\circ$ against the propagation direction of the GW, as sketched in Fig. 1. The coordinate transformation between the $(+)$polarisation and the (*x*, *y*)-oriented arms leads to a halving of the GW amplitude contribution. Additionally, the trajectory of light changes over the duration of the gravitational wave, i.e. we replace in Eq. (1) $h_+(t)$ by $h_+\!\left( t+y/\sqrt{2c^2} \right) /2$ for the light propagating along the *y* axis. If we carry out the calculation analogous to the one above, we arrive at

7 $$\begin{aligned} \Delta \tau _{2L}(t) = -\frac{h_{+}}{2c}\int \limits _0^{L} \cos \!\left[ 2\pi f\! \left( t - \frac{2 L}{c}+\frac{y'(1+1/\sqrt{2})}{c}\right) \right] dy' + \frac{h_{+}}{2c}\int \limits _L^0 \cos \!\left[ 2\pi f\! \left( t+\frac{y'(1-1/\sqrt{2})}{c}\right) \right] dy' \, . \end{aligned}$$

For the specific case of $f\!\ll \!f_\textrm{FSR}$ we obtain

8 $$\begin{aligned} |\Delta \tau _{2L}| \approx \frac{Lh_+}{2c} \;\;\;\Rightarrow \;\;\; \Delta L \approx \frac{Lh_+}{4} \, , \end{aligned}$$

i.e. half of the signal for the optimal alignment according to Eq. (6). However, for the specific case of $f = f_\textrm{FSR}$ we obtain

9 $$\begin{aligned} \!\!|\Delta \tau _{2L}| \approx \frac{L}{5c} h_+ \;\;\;\Rightarrow \;\;\; \Delta L \approx \frac{Lh_+}{10} \, , \end{aligned}$$

which is just five times worse than the response at low frequencies according to Eq. (6). Similarly, the detector has significant response at all frequencies corresponding to $f = n \cdot f_\textrm{FSR}$, see next section.

Current GW observatories have optical responses to gravitational waves according to Eqs. (5)-(9). Since our calculation uses the TT-gauge, the light’s red-shift (when propagating through expanding spacetime) and blue-shift (when propagating through shrinking spacetime) are already included^32^. We note that we have set the phase of the GW to zero. In the more general case, the above equations would get slightly more complex, as we show below.

## Frequency combs of high sensitivity of current GW observatories

An optical resonator shows a longitudinal resonance if its round trip length 2*L* equals an integer multiple of the wavelength of the light coupled to it ($2L = n \cdot \lambda$). The frequency spacing of two neighboring resonances is called the free spectral range ($f_\textrm{FSR} = c/2L$). Phase modulations of carrier light that meets one resonance condition are optically enhanced at all frequencies *f* that correspond to $n \cdot f_\textrm{FSR}$, where *n* is again a natural number. In the case of the 4 km LIGO detectors, the comb spacing is 37.5 kHz. The 3 km Virgo and KAGRA detectors have a comb spacing of 50 kHz. The 1.2 km long, folded-arm signal recycling cavity of GEO 600 results in integer multiples of 125 kHz. In all cases, the linewidths of the resonances are of the order of a kHz. The proposed 10 km Einstein Telescope and the 40 km Cosmic Explorer have comb spacings of approximately 15 kHz and 3.75 kHz^27^. Similarly, LISA has a comb spacing of $\approx 20$ mHz^37^.

**Figure 2:**
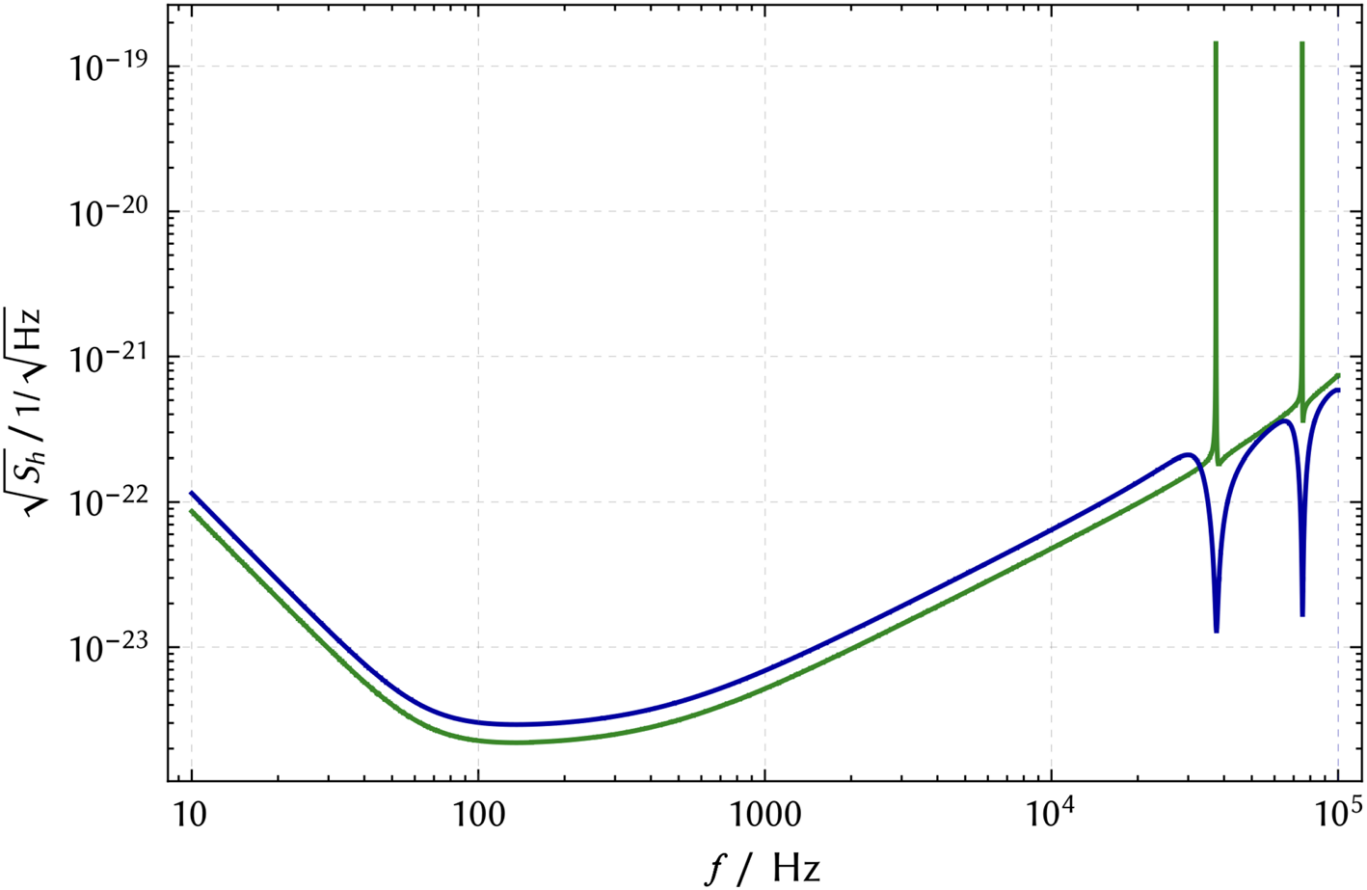
Strain-normalized quantum noise amplitude spectral densities of Advanced LIGO according to its design values (without quantum noise squeezing^35,36^) for two different sky locations of the source, averaged over $(+)$ and $(\times )$ polarisations of the GW. Lowest noise at audio band frequencies is achieved for the zenith sky location (green), which the inverse of Eq. (5) refers to. The second curve (blue) refers to a sky location that minimizes the noise to signal ratio at LIGO’s first “FSR frequency” of 37.5 kHz ($\theta = 0.767, \phi = 0.241$).

Spectral sensitivities of GW observatories are best described by one-sided (positive frequencies only) amplitude noise spectral densities (ASD) normalized to the signal strength at the respective frequency. The relation between the ASD normalized to strain *h* and normalized to the round trip phase difference of the laser beams $\varphi$ reads

10 $$\begin{aligned} \sqrt{S_{h}(f)} = \frac{c}{2L\omega } \sqrt{S_{\varphi }(f)} \; , \end{aligned}$$

where $\omega$ is the angular frequency of the laser light. If the noise in $S_\varphi (f)$ is dominated by photon shot noise^36^, which is typically a justified approximation for signal frequencies above a few kilohertz, the noise spectrum alone is “white”, i.e. independent of the frequency. The phase signal on the laser light, however, depends on the frequency and additionally on the GW’s polarisation with respect to the orientation of the observatory, on its alignment with respect to the GW’s direction of propagation, and on length and linewidth of the arm resonators and further enhancement resonators coupled to it. We show how the phase signal for an observatory with two equally long arms under 90$^\circ$ can be calculated for arbitrary alignments in the next section.

Fig. 2 presents quantum noise amplitude spectral densities of Advanced LIGO for two different sky locations of the GW sources for mixed GW polarisation. The zenith sky location provides the lowest noise for audio-band frequencies (green). The second sky location is optimized for lowest noise at LIGO’s FSR frequency of 37.5 kHz (blue). LIGO’s quantum noise limited amplitude sensitivity at this frequency is just a factor of about six lower than that at the optimal frequency between 100 and 200 Hz.

**Figure 3:**
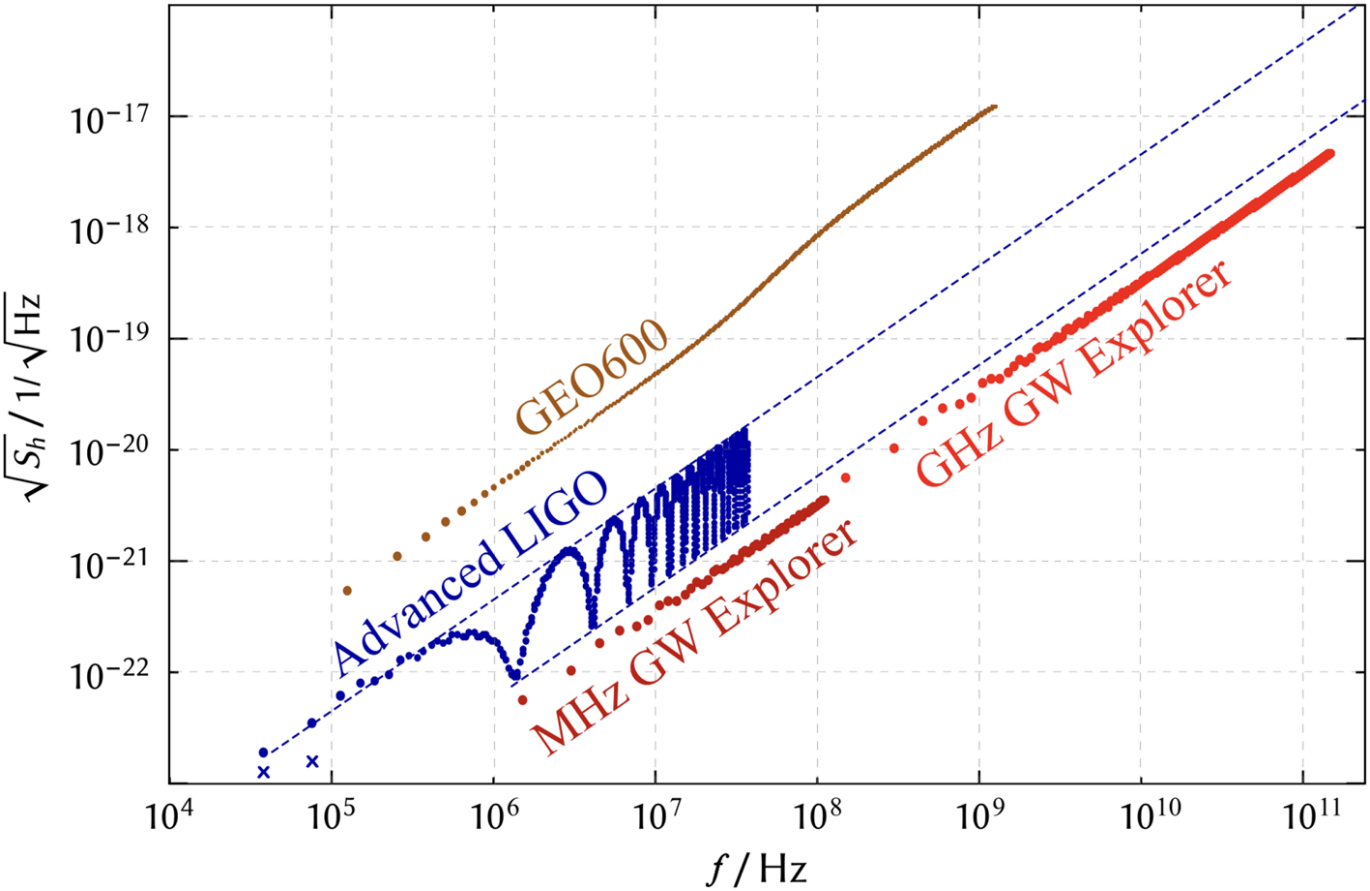
Polarisation-averaged and sky-averaged amplitude shot-noise spectral densities at frequencies $n\!\cdot \!f_\textrm{FSR}$. GEO 600 for up to $n=10^4$: folded 1200-m arms without arm resonators with 1.5 kW light power in each arm, also featuring power recycling and signal recycling resonators and 6 dB quantum noise squeezing^38,39^ up to about 50 MHz^40^. For the Advanced LIGO, the two crosses correspond to the minima in Fig. 2. The noise to signal ratio further drops with a frequency spacing of about 3 MHz. This additional comb corresponds to the FSR of the signal extraction cavity coming on resonance. (The distance between the arm resonator input mirrors and the signal extraction mirror, which is not shown in Fig. 1, is approximately 55 m). Dashed lines are the extrapolated envelopes. Also shown is our result for two potential small-scale laser interferometers which we name “MHz-GW explorer” ($n\!\le \!63$) and “GHz-GW explorer” ($n\!\le \!10^3$): 100-m and 1-m arm resonators, respectively, with 10 MW optical power at 1550 nm, input mirror reflectivity of 99.995% and 10 ppm round trip loss, and no signal recycling cavity.

Fig. 3 presents polarisation-averaged and sky-averaged amplitude shot-noise spectral densities at the “FSR frequencies” of two GW detectors in operation and two conceivable detectors with less complexity and shorter arms. In all traces, the noise to signal ratio increases proportional to the GW frequency. This is a general property of laser interferometers with resonator round trip time larger than the gravitational wave period, because the effective propagation time over which the effect of the GW is accumulated is inversely proportional to the GW frequency. This property is described by the sinc-function in Eq. (5) and in more general expressions discussed in the next section. Comparing the interferometer sensitivities shows that for frequencies above the largest FSR (here 150 MHz), arm length is not an issue.

Fig. 3 also shows the equivalent spectral densities for simple Michelson laser interferometers enhanced with just arm resonators of 100 m and 1 m lengths, our “MHz GW explorer” and “GHz GW explorer”, respectively. The curves assume light powers of 10 MW in each arm resonator, a coupling mirror reflectivity of 99.995% and 10 ppm round trip loss. Light with a squeezed quantum uncertainty is not assumed.

**Figure 4:**
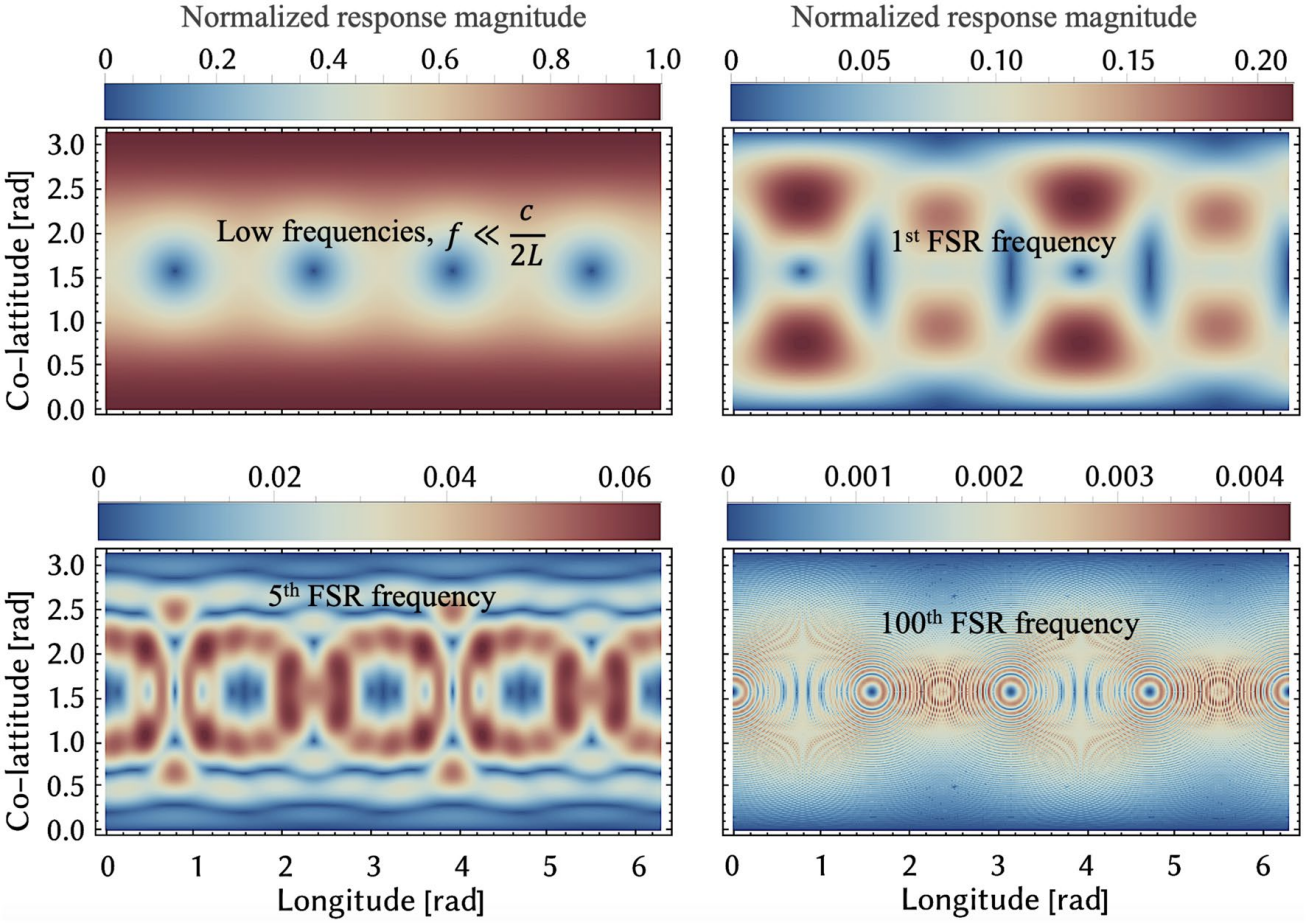
Antennae patterns for detecting gravitational waves of a mixed polarisation at different FSRs: 0th, 1st, 5th and 100th. It can be seen that while for the 0th FSR the maximal response corresponds to the GWs coming from zenith, for higher FSRs the response there is zero.

## High-${{\varvec{f}}}\!$ antennae pattern

Here, we outline how to derive the direction-dependent amplitude of GW induced phase signals for an interferometric GW observatory with perpendicular arms following the work^33,34^. The arms are oriented along *x* and *y*, of which the *x*-arm provides the reference. We define the GW-induced change of the optical round-trip phase as

11 $$\begin{aligned} \varphi _a = h_a\mathcal {F}_a(f)G(f) \, , \end{aligned}$$

where $h_a$ is the polarisation of the GW coming from direction *a* defined with respect to the *x*-arm, $\mathcal {F}_a(f)$ is the response to this GW, and *G*(*f*) is the optical transfer function (e.g. of an arm cavity). Direction *a* then reads

12 $$\begin{aligned}&a_x = \sin \theta \sin \phi \, , \end{aligned}$$

13 $$\begin{aligned}&a_y = \sin \theta \cos \phi \, , \end{aligned}$$

where $\theta \in [0,\pi ]$ is co-latitude, and $\phi \in [0, 2\pi ]$ is longitude of the source. The polarisation along the two arms is related to $(+,\times )$polarisation through the coordinate transformation

14 $$\begin{aligned} h_{xx}(\theta , \phi ) =&h_{+} (\cos ^2 \theta \cos ^2\!\phi - \sin ^2\!\phi ) +2 h_{\times } \cos \theta \sin \phi \cos \phi , \end{aligned}$$

15 $$\begin{aligned} h_{yy}(\theta , \phi ) =&h_{+} (\cos ^2\!\theta \sin ^2\!\phi - \cos ^2\!\phi ) -2 h_{\times } \cos \theta \sin \phi \cos \phi \, . \end{aligned}$$

Following the detailed derivation of the high-frequency response given in ^33,34^, we define the response function

16 $$\begin{aligned} \mathcal {F}_a(f) = \frac{e^{-i\pi f/f_\textrm{FSR}}}{2(1 - a^2)\pi f/f_\textrm{FSR}} \times \big \{ \sin (\pi f/f_\textrm{FSR}) - a \sin (\pi a f/f_\textrm{FSR}) - i a \cos (\pi f/f_\textrm{FSR}) - \cos (\pi f a/f_\textrm{FSR}) \big \} \, . \end{aligned}$$

For gravitational waves from zenith ($\theta = 0$), the response is reduced to the sinc function, as in Eq. (5), and turns zero for $f=f_\textrm{FSR}$. For other orientations, however, the above equation is not zero for $f=f_\textrm{FSR}$.

The phase difference between two arms can be expressed in terms of the response function

17 $$\begin{aligned} \Delta \varphi = \varphi _x - \varphi _y = h_{xx}\mathcal {F}_x(f)G_x(f) - h_{yy}\mathcal {F}_y(f)G_y(f) \,, \end{aligned}$$

where $\mathcal {F}_{x,y} = \mathcal {F}_a|_{a=a_x,a_y}$. Typically, the optical response of the arms is identical, $G_x(f)=G_y(f)=G(f)$. Importantly, in detectors with symmetric arms, the response to GWs only depends on the arm lengths, and not on other parameters of optical responses, which is reflected in the fact that *G*(*f*) enters as a common factor.

We can re-write the phase difference in terms of the response of the detector to the two polarisations yielding

18 $$\begin{aligned} \Delta \varphi = \left( h_{+}\mathcal {F}_+(f) - h_{\times }\mathcal {F}_{\times }(f)\right) G(f), \end{aligned}$$

where $\mathcal {F}_{+}$ can be computed from this equation by substituting Eqs. (14, 15) into (17) and formally setting $h_{+}=1, h_{\times }=0$ (and vice versa for $\mathcal {F}_{\times }$):

19 $$\begin{aligned} \mathcal {F}_{+}(f) =&\;\mathcal {F}_{x}(f) (\cos ^2 \theta \cos ^2\!\phi - \sin ^2\!\phi ) - \mathcal {F}_{y}(f) (\cos ^2\!\theta \sin ^2\!\phi - \cos ^2\!\phi ), \end{aligned}$$

20 $$\begin{aligned} \mathcal {F}_{\times }(f) =&\;2 \mathcal {F}_{x}(f) \cos \theta \sin \phi \cos \phi + 2 \mathcal {F}_{y}(f) \cos \theta \sin \phi \cos \phi \, . \end{aligned}$$

It is common to use the averaged polarisation response defined by

21 $$\begin{aligned} \bar{\mathcal {F}} = \sqrt{|\mathcal {F}_{+}|^2 + |\mathcal {F}_{\times }|^2 }. \end{aligned}$$

To quantify the sensitivity of a GW observatory, it is also common to use the sky-averaged response, where the response is integrated over the whole sky. We have followed both conventions in Fig. 3.

Alternatively, the signal amplitude can be displayed for a fixed signal frequency as a function of the localisation in the sky, which are the so-called antennae pattern. Fig. 4 show the characteristic antennae pattern of GW observatories with perpendicular equally log arms for the audio band and a selection of their FSR frequencies. The upper plot presents the response to audio band frequencies. It is maximal for GWs coming from zenith and nadir. The next plots present the antennae pattern for frequencies $n \!\cdot \! f_\textrm{FSR}$, with $n = 1, 5, 100$. Here, the highest response at FSR frequencies is achieved for other sky locations. The higher the frequency, the finer the antenna pattern. The maximum response is inversely proportional to the order *n* of the FSR.

## Discussion and conclusion

Fig. 3 shows points of noise spectral density minima of resonator amplified laser interferometers with moving test mass mirrors and reveals a significant optical sensitivity for gravitational waves at higher kHz, MHz and GHz frequencies. There are no modifications required to the optical systems of existing GW observatories and GW observatory prototypes for their use in the high-frequency range. However, the procedure for absolute calibration of the detector response would have to be adapted, as discussed in e.g.^28^. In addition, faster photodetector electronics with low dark noise in the high frequency range would be required. Systems have been demonstrated up to several GHz^44–46^. Significantly higher frequencies have been achieved with upstream optical parametric amplification^47^. Finally, the data acquisition and control systems would have to be adapted to the higher frequencies. For example, some attention will have to be paid to the coupling of laser frequency noise^42^ as well as high-frequency thermal noise of the test mass mirrors^43^. If the one-MHz range was read out by the currently operating Advanced LIGO, a sensitivity of the order $10^{-22}/ \sqrt{\rm Hz}$ would be achieved, which is well inside the range of existing and proposed dedicated instruments in table 1 of Ref. ^22^ as well as haloscopes used to search for dark matter axions, but also potentially sensitive to GWs, as presented in figure 7 of Ref. ^48^.

We would like to point out another approach that allows detecting high-frequency GWs with existing observatories: the nonlinear GW memory^23,24,52–54^. This effect is a permanent expansion of the distance between two free-falling test masses left after a passing burst of GWs. The expansion contains frequency components at frequencies significantly lower than those of the primary GW signal, but with comparable amplitudes. Fig. 3 in^55^ demonstrates that a sine-Gaussian burst signal around 100 MHz that would appear with the signal-to-noise ratio (SNR) of 5 in a proposed high-frequency detector with a broadband sensitivity of $10^{-22}/ \sqrt{\rm Hz}$ ^56^ would produce an audio-band memory signal with SNR exceeding 1000 in Advanced LIGO. On the similar grounds, Ref. ^57^ points out that if the observation in^58^ at the frequency of 5.5 MHz came from a GW, the LIGO/Virgo detectors would have registered the memory of this signal with an SNR of greater than $10^6$ in the audio band. The search of GW memories from merging supermassive black holes is commonly performed using pulsar timing arrays^59–62^. The primary signals from these mergers appear in the frequency band from $\mu$Hz up to Hz. The memory signal, however, would be observable at nHz frequencies, where pulsar timing arrays have their highest sensitivity to GWs^16^.

These two examples demonstrate that the existing GW observatories are sensitive to high-frequency GWs, even if they are not directly designed for this purpose. Moreover, their sensitivity is comparable to that of the proposed dedicated high-frequency GW detectors. The currently operating Advanced LIGO has a photon shot noise amplitude spectral density normalized to GW strain of the order $10^{-22}/ \sqrt{\rm Hz}$ around one MHz, which is well inside the range of existing and proposed dedicated instruments, compare table 1 in^22^. Our result can also be directly compared with Fig. 7 in Ref. ^48^, which shows the amplitude spectral densities of several axion detectors for measuring GWs in the frequency band from about $10^4$ to over $10^{10}$ Hz. Overall, this highlights the necessity of re-evaluating whether it is essential to invest in the development of novel detectors for the high-frequency range.

## Data Availability

The data that support the plots within this paper and other findings of this study are available from the corresponding author upon reasonable request.

## Declarations

### Code availability

The code that supports the findings of this study is available at https://doi.org/10.5281/zenodo.15054036.
